# Inspecting the Dangers of Feeling like a Fake: An Empirical Investigation of the Impostor Phenomenon in the World of Work

**DOI:** 10.3389/fpsyg.2016.01445

**Published:** 2016-09-27

**Authors:** Mirjam Neureiter, Eva Traut-Mattausch

**Affiliations:** Economic and Organizational Psychology, Department of Psychology, University of SalzburgSalzburg, Austria

**Keywords:** impostor phenomenon, career adaptability, career optimism, job market knowledge, salary, organizational citizenship behavior, commitment

## Abstract

To investigate the link between the impostor phenomenon (IP), career self-management (CSM) factors, and work-relevant outcomes, we looked at the IP's impact on career optimism, career adaptability, and knowledge of the job market, as well as on employee- and organizationally-relevant outcomes. We analyzed data from 238 working professionals (57% female) using parallel multiple mediation analyses. The results revealed that the IP was negatively related to all work-relevant outcomes through decreased CSM factors, which were subsequently associated with the outcomes. As hypothesized, employee-relevant subjective outcomes were mediated by optimism and employee-relevant objective (i.e., economic) outcomes by adaptability and knowledge. Additional mediating effects occurred. Regarding organizationally relevant outcomes, adaptability mediated the IP's impact on organizational citizenship behavior. The IP was only indirectly related to continuance commitment through adaptability and to affective commitment through optimism. We discuss the theoretical and practical implications and offer ideas for future research.

*Many skilled, accomplished executives fear that they're not good enough—impostors who are bound to be found out. By undervaluing their talent, are they ruining their careers and companies?* (Kets de Vries, 2005, p. 108).

## Introduction

The impostor phenomenon (IP) is especially interesting in the world of work as it concerns high-achieving individuals who instead of being proud of their success, experience intense feelings of intellectual and/or professional fraudulence (Clance and Imes, 1978). Despite objective evidence to the contrary, such as professional advancements, positive reviews, or honors and awards, they are unable to internalize their successful experiences (e.g., Bernard et al., 2002; Want and Kleitman, 2006). Sufferers of the IP experience discrepancies between other's perceptions of them as being successful and their own perceptions as being deficient (Clance and Imes, 1978). They perceive perpetual overestimation of their abilities by supervisors and colleagues and fear being exposed as “impostors” (Clance et al., 1995). Whereas other employees experience growth in self-esteem after achieving success at work, IP sufferers experience an increase in their sense of fraudulence (see also “the impostor cycle”; Clance, 1985), negative feelings, and dissatisfaction (Cowman and Ferrari, 2002). The IP is fueled by low self-esteem and the fear of failure as well as of success, and it acts as an inner barrier to career development (Jöstl et al., 2012; Neureiter and Traut-Mattausch, 2016). However, attention to the IP explicitly in the world of work is just beginning to emerge and remains sparse. Researchers have pointed out the need to investigate the consequences of the IP for both employees and organizations (Whitman and Shanine, 2012). By answering this call, we hope to complement existing studies with student samples and health-related outcomes with our investigation of working professionals and work-relevant outcomes.

### Employee-relevant outcomes

In the domain of careers, studies have shown that the more impostor feelings individuals have, the less career planning they report (Neureiter and Traut-Mattausch, 2016). Further, the IP has been identified as an integrative phenomenon that functions as an inner barrier to moving up to higher occupational levels and leadership positions (Neureiter and Traut-Mattausch, 2016). Moreover, the IP has been found to be negatively related to job satisfaction (Vergauwe et al., 2015). Vergauwe et al. (2015) considered job satisfaction because it is one of the most predominant outcome variables in the applied literature (Judge and Kammeyer-Mueller, 2012) and has been shown to be related to a range of important constructs including employee well-being (Faragher et al., 2005) and performance (Judge et al., 2001). *Career* satisfaction extends beyond job satisfaction as it concerns not only a person's current job but also that person's entire career. As Jawahar and Stone (2015, p. 6) stated, “career satisfaction measures the extent to which individuals believe their career progress is consistent with their goals, values, and preferences.”

Other important employee-relevant outcomes include perceived internal and external marketability and other-referent subjective career success. Perceived internal marketability represents the belief that one's own employer considers one to be a valuable employee. By contrast, perceived external marketability is the perception that one is valuable to other companies and so across organizational boundaries (Eby et al., 2003). Other-referent subjective career success concerns perceived career success in comparison with others such as colleagues or peers and has been shown to explain unique variance in overall subjective career success (Heslin, 2003, 2005). Given that a negative relation between the IP and job satisfaction has been established (Vergauwe et al., 2015), we would also expect a negative relation of the IP to these other employee-relevant outcomes.

The outcomes discussed above primarily concern subjective perceptions (= subjective outcomes) and have been used as indicators for subjective career success (Spurk and Volmer, 2013). Another kind of employee-relevant outcomes involves more economic concerns (= economic outcomes), that is, promotions and salary, two measures of objective career success (Spurk and Volmer, 2013). Impostors, who are not aware of their own competences (Bernard et al., 2002) and who have low self-esteem (e.g., Sonnak and Towell, 2001), are assumed to have difficulties in requesting a high salary. The IP also functions as an inner barrier to promotions: Impostors show less career striving within their organization and less motivation to take a leading position (Neureiter and Traut-Mattausch, 2016). Hence we would expect a negative relation to those employee-relevant economic outcomes, as well.

**Table:** 

**Hypothesis 1:**	There is a negative relation between the IP and employee-relevant subjective outcomes.
**Hypothesis 2:**	There is a negative relation between the IP and employee-relevant economic outcomes.

### Organizationally relevant outcomes

The IP has also been found to reduce more organizationally relevant outcomes, namely, organizational citizenship behavior (OCB) and affective commitment, and to increase continuance commitment (Grubb and McDowell, 2012; Vergauwe et al., 2015). OCB is described as “individual behavior that is discretionary, not directly or explicitly recognized by the formal reward system, and in the aggregate promotes the efficient and effective functioning of the organization” (Organ et al., 2006, p. 3). To cover their perceived fraudulence, impostors are likely to work hard to fulfill the demands of in-role behavior according to their own high standards. As personal resources are restricted, this will result in less extra-role behavior, such as OCB. For example, impostors would never volunteer to brief new colleagues because they do not have enough resources for it.

The IP has been found to be related to organizational commitment (Grubb and McDowell, 2012; Vergauwe et al., 2015). In general, organizational commitment can be broken down into three components: affective, normative, and continuance commitment (Allen and Meyer, 1990). We focus on affective and continuance commitment, as previous research has already established an impact of the IP on these components (Grubb and McDowell, 2012; Vergauwe et al., 2015). For instance, Vergauwe et al. (2015) found that impostors were more engaged in continuance commitment and explained this as stemming from a perceived lack of alternative employment opportunities, which has been found to be positively related to continuance commitment (Powell and Meyer, 2004). On the other hand, impostor feelings foster the conviction that the impostor cannot stay at his or her company long term, which in turn may lead to less affective commitment (Grubb and McDowell, 2012).

**Table:** 

**Hypothesis 3:**	There is a negative relation between the IP and organizationally relevant outcomes.

A summary of the studies investigating the IP in the world of work could be found in Table 1. In sum, the IP seems to pose a considerable risk to an employee's career and may well have negative consequences for companies. However, the question of what process variables are responsible for these negative effects of the IP on employee- and organizationally-relevant outcomes remains open. Therefore, we furthermore focused on identifying the factors that play a role in the mentioned processes.

**Table 1:** **Summary of studies investigating the impostor phenomenon and work-relevant variables**.

**Study**	**Sample(s)**	**Work-relevant outcome variables**	**Effects**
Jöstl et al., 2012	631 (62% female) doctoral students	Research self-efficacy	−0.09^*^
McDowell et al., 2015	588 (33% female) university staff	Self-efficacy	−0.36^**^
		Perceived organizational support	−0.10^*^
Grubb and McDowell, 2012	588 (33% female) university staff	Organizational citizenship behavior	−0.32^***^
		Affective commitment	−0.11^**^
		Continuance commitment	0.20^***^
Vergauwe et al., 2015	201 (58% female) employees	Job satisfaction	−0.29^***^
		Organizational citizenship behavior	−0.35^***^
		Affective commitment	−0.11
		Continuance commitment	0.22^**^
Bechtoldt, 2015	190 (35% female) managers	Biased task-delegation decisions	0.10–0.22^*^
Neureiter and Traut-Mattausch, 2016	212 (70% female) employees	Career planning	−0.23^***^
		Non-observable career striving	−0.12
		Observable career striving	0.14^*^
		Motivation to lead	−0.19^**^
Neureiter and Traut-Mattausch, 2016	110 (50% female) employees	Career planning	−0.76^***^
		Career striving	−0.51^***^
		Motivation to lead	−0.58^***^

* *p < 0.05*,

** *p < 0.01*,

*** *p < 0.001*.

### Career self-management factors

Such crucial process variables could be found by looking at variables that have already been demonstrated to foster the desired outcomes. Researchers have identified the so-called career self-management (CSM) factors as being helpful in attaining high work-relevant outcomes (McIlveen et al., 2012; Spurk and Volmer, 2013). Ng and Feldman (2014) concluded that “individuals' CSM and its impact on subjective career success become increasingly prominent concerns for organizations and employees alike” (p. 177). More specifically, the CSM factors *career optimism, career adaptability*, and *knowledge of the job market* have been shown to have positive effects on work-relevant outcomes such as job satisfaction and promotions in working professionals (Spurk and Volmer, 2013). Hence, investigating these factors could provide further insights into the process that is responsible for the negative effects of the IP on employee- and organizationally-relevant outcomes. Moreover, all of them are predestinated to be reduced by the IP.

Career optimism (Scheier et al., 1994; Carver and Scheier, 2012) has been shown to have a beneficial impact on work productivity (Seligman and Schulman, 1986) as well as on other desirable characteristics such as happiness, achievement, and perseverance (Peterson, 2000). Moreover, optimism has positive effects on career planning and exploration and on confidence in career decisions, as well as on the level of career-related goals (Creed et al., 2002). Previous research found that employees with higher career optimism also had higher career and job satisfaction, higher perceived internal and external marketability, and higher other-referent subjective career success (Spurk and Volmer, 2013). Furthermore, career optimism explained variance in career satisfaction beyond other relevant career variables and personality. Optimistic individuals are assumed to “expect the best possible outcome or to emphasize the most positive aspects of [their] future career development, and [be comfortable] performing career planning tasks” (Rottinghaus et al., 2005, p. 11). Different to those optimistic individuals, impostors report various negative thoughts and emotions, and they are disposed to feelings of depression (Chrisman et al., 1995; Thompson et al., 1998; Bernard et al., 2002; Oriel et al., 2004; McGregor et al., 2008). Moreover, even if impostors are successful one time, they remain fearful of failing the next time and of being discovered as a fake (see also “the impostor cycle”; Clance, 1985), what prevents them from developing an optimistic future perspective. Based on these findings, we expect the IP to be negatively related to optimism. Hence, if the IP reduces career optimism, which has been found to benefit efforts to gain high employee-relevant subjective outcomes, we expect that less career optimism mediates this relation.

**Table:** 

**Hypothesis 4:**	The negative relation between the IP and employee-relevant subjective outcomes is mediated by less career optimism.

Career adaptability was identified in the theory of career construction (e.g., Savickas, 1997), where it was defined as “the readiness to cope with the predictable tasks of preparing for and participating in the work role and with the unpredictable adjustments prompted by changes in work and working conditions” (p. 254). Rottinghaus et al. (2005) defined it as “the way an individual views his or her capacity to cope with and capitalize on change in the future, level of comfort with new work responsibilities, and ability to recover when unforeseen events alter career plans” (p. 11). Savickas and Porfeli (2012) suggested that career adaptability can be seen as self-regulation strengths or capacities that a person may draw upon to solve career-related tasks. Career adaptability has been shown to be negatively related to perceived internal barriers (Soresi et al., 2012; Urbanaviciute et al., 2016). The IP has already been identified as such an internal barrier in the context of career development as more impostor feelings prevented employees from striving for higher career levels within their organization (Neureiter and Traut-Mattausch, 2016). Moreover, impostor feelings were shown to be negatively related to career adaptability in students (Neureiter and Traut-Mattausch, submitted). Consequently, we expect a negative relation between the IP and career adaptability in working professionals as well. The more impostor feelings an individual has, the fewer adaptability resources will be present. However, high adaptability is needed to enhance employee-relevant economic outcomes (Spurk and Volmer, 2013).

Knowledge of the job market has explained additional variance beyond other relevant career variables and personality (Spurk and Volmer, 2013). Knowledge of the job market refers to “how well an individual understands job market and employment trends” (Rottinghaus et al., 2005, p. 11). It is seen as an extended construct of career exploration (Spurk et al., 2015) that has been shown to be positively related to career growth and success (Zikic et al., 2006). Impostors are convinced that they get a job because they have been lucky or in the right place at the right time (Clance and Imes, 1978), so why should they show career exploration or expand their job market knowledge? As they have an external locus of control (Thompson et al., 2000; Sightler and Wilson, 2001) and only little self-efficacy (Vergauwe et al., 2015), they might not see any demands for exploring career options or updating their job market knowledge. Consequently, we expect that the IP has a negative influence on job market knowledge and on career adaptability, CSM factors that would be needed to enhance employee-relevant economic outcomes such as salary or number of promotions.

**Table:** 

**Hypothesis 5:**	The negative relation between the IP and employee-relevant economic outcomes is mediated by less career adaptability and knowledge of the job market.

Besides influencing employee-relevant outcomes, the IP has been shown to impact organizationally relevant outcomes, as well. Impostors showed less OCB and affective commitment, as well as more continuance commitment (Grubb and McDowell, 2012; Vergauwe et al., 2015). Hence, there is preliminary evidence that the IP will further have a negative impact for organizations. However, as impostors are high-achieving individuals who are often integrated in succession planning (Parkman and Beard, 2008), human resources managers should support them when required. To prevent negative developments and provide appropriate support it is important to identify how impostor feelings shape an employee's attitudes in the workplace (Whitman and Shanine, 2012), which in turn affects employee- and organizationally-relevant outcomes. As CSM factors are of great concern for employees and organizations alike (Ng and Feldman, 2014), we investigate their role regarding organizationally relevant outcomes, as well.

That impostor feelings may reduce adaptability could explain the negative relation to OCB, as well. Adaptability resources are needed to handle new situations or unforeseen work-related tasks such as the need to brief a new colleague. As argued earlier, the IP reduces adaptability and impostors will not show any voluntary initiative. Consequently, we expect that the negative relation between the IP and OCB can be explained through less career adaptability.

**Table:** 

**Hypothesis 6:**	The negative relation between the IP and OCB is mediated by less career adaptability.

Obtaining alternative employment goes along with adaptability resources. Adaptive behavior in the form of career exploration was found to be positively related to turnover intentions, job-search behaviors, and actual turnover and negatively related to loyalty (Klehe et al., 2011). As impostor feelings are assumed to reduce adaptability, impostors tend not to leave their employers and will engage in continuance commitment to retain their current position. On the other hand, impostor feelings may lead to less career optimism, which will result in the pessimistic assumption that they could not stay at their place of employment in the long term, leading to less affective commitment (Grubb and McDowell, 2012).

**Table:** 

**Hypothesis 7:**	The positive relation between the IP and continuance commitment is mediated by less career adaptability.
**Hypothesis 8:**	The negative relation between the IP and affective commitment is mediated by less career optimism.

## Methods

### Participants and procedure

We contacted working professionals by e-mail with addresses provided by the university's alumni club and through personal contacts. E-mail recipients were invited to complete an online questionnaire containing measures of the IP, the Career Futures Inventory (CFI), and measures of the work-relevant outcomes (employee-relevant outcomes, OCB, continuance, and affective commitment). The study took place in Austria (Europe). It was approved by the ethics board of the university and carried out in accordance with their recommendations. All participants gave informed consent in accordance with the ethical standards of the American Psychological Association (APA). The participants were informed about the voluntary nature of participation and the confidential use of data. They were further informed that there were no right or wrong answers to the questions and that drawing any personal inferences from them would not be possible. To assure anonymity, participants were not asked for information that could identify them (e.g., names) on the questionnaire. Participants were free to withdraw at any time. Participants were also provided with the name and e-mail address of the responsible investigators. In all, 361 people opened the link, of which 238 (57% female, 43% male; *M*_age_ = 37.62 years, *SD* = 11.43) completed the questionnaire and 123 dropped out, representing a response rate of about 66%. The mean work experience was 14.81 years (*SD* = 11.10). The biggest proportion of participants (45%) reported working for an employer with more than 250 employees. Seventeen percent worked for an employer with 50–249 employees, 20% with 10–49 employees, and 18% with fewer than 10 employees. Sixty percent reported having one of the following academic degrees: 18% bachelor's, 8% teacher training, 52% master's, and 22% Ph.D. Filling out the online survey took on average 19 min, 42 s (median: 16 min, 22 s). The participants did not receive any compensation for their participation in the study.

### Measures

The Cronbach's alphas as well as the means, standard deviations, and correlations of the main variables can be found in Table 2. Unless otherwise stated, all measures used a 5-point scale ranging from 1 (*strongly disagree*) to 5 (*strongly agree*). The responses were coded such that low values represent low instances of the participants' perception of each construct.

**Table 2:** **Means, standard deviations, and correlations for the main variables**.

**Variable**	***M***	***SD***	**1**	**2**	**3**	**4**	**5**	**6**	**7**	**8**	**9**	**10**	**11**	**12**	**13**	**14**
1 Impostor feelings	2.23	0.88	(0.90)													
2 Career satisfaction	4.02	0.77	−0.29^***^	(0.88)												
3 Job satisfaction^a^	5.40	1.09	−0.27^***^	0.53^***^	–											
4 Other-referent subjective career success^b^	3.75	0.81	−0.34^***^	0.61^***^	0.34^***^	(0.67^***^)										
5 Internal marketability^b^	4.08	0.83	−0.28^***^	0.46^***^	0.41^***^	0.40^***^	(0.76^***^)									
6 External marketability	3.43	0.90	−0.15^*^	0.41^***^	0.23^***^	0.41^***^	0.38^***^	(0.79)								
7 Salary	8.20	4.33	−0.25^***^	0.21^***^	0.23^***^	0.35^***^	0.21^**^	0.27^***^	–							
8 Promotions^c^	2.46	1.85	−0.13^*^	0.25^***^	0.16^*^	0.24^***^	0.19^**^	0.23^***^	0.53^***^	–						
9 Organizational citizenship behavior^a^	5.39	1.17	−0.38^***^	0.21^**^	0.19^**^	0.14^*^	0.17^**^	0.18^**^	0.16^*^	0.19^**^	(0.74)					
10 Continuance commitment	3.24	1.05	0.06	−0.00	0.11	−0.00	−0.05	−0.23^***^	−0.02	−0.06	−0.15^*^	(0.73)				
11 Affective commitment	3.71	0.98	−0.08	0.28^***^	0.56^***^	0.11	0.35^***^	0.02	0.11	0.07	0.09	0.33^***^	(0.90)			
12 Optimism^d^	4.23	1.07	−0.36^***^	0.60^***^	0.37^***^	0.57^***^	0.37^***^	0.39^***^	0.13	0.13^*^	0.20^**^	−0.09	0.19^**^	(0.87)		
13 Adaptability^d^	4.60	0.88	−0.34^***^	0.37^***^	0.23^***^	0.36^***^	0.29^***^	0.44^***^	0.27^***^	0.27^***^	0.31^***^	−0.26^**^	0.02	0.46^***^	(0.90)	
14 Knowledge of the job market^d^	3.07	1.17	−0.30^***^	0.15^*^	0.04	0.28^***^	0.12	0.33^***^	0.30^***^	0.22^**^	0.10	−0.11	−0.02	0.25^***^	0.38^***^	(0.75)

N = 238 (57% female, 43% male). Entries in parentheses on the diagonal are Cronbach's alpha reliability coefficients.

a *Measured on a 7-point scale*;

b *items were correlated*;

c *mean and standard deviations of employees who reported promotions*;

d *measured on a 6-point scale*;

* *p < 0.05*,

** *p < 0.01*,

*** *p < 0.001*.

#### Impostor phenomenon

Impostor feelings were assessed using the 20-item German-language version of the Clance Impostor Phenomenon Scale originally developed by Clance (1985) and Klinkhammer and Saul-Soprun (2009)^1^. One example item is “I can give the impression that I'm more competent than I really am.”

#### Work-relevant outcomes

Employee-relevant subjective outcomes were measured through career and job satisfaction, internal and external marketability, and other-referent subjective career success. Career satisfaction was measured with the five-item scale from Greenhaus et al. (1990); sample item: “I am satisfied with the progress I have made toward meeting my overall career goals”). One 7-point-scale item was used for measuring general job satisfaction: “Thinking about all the things that are important for your work, how satisfied are you?” (Wanous et al., 1997). Perceived internal and external marketability were each assessed with three items (e.g., for internal marketability: “My company views me as an asset to the organization”; for external marketability: “Given my skills and experience, other organizations view find me as a value-added resource”; (Eby et al., 2003). Other-referent subjective career success was assessed with two items: “How satisfied are you with the professional advancement you have attained relative to your former fellow students?” and “How satisfied are you with the professional advancement you have attained relative to your co-workers?” (Heslin, 2003, 2005; Abele et al., 2010). Employee-relevant economic outcomes were measured using two indicators, namely, salary class and number of promotions (e.g., Ng et al., 2005; Abele et al., 2010; Spurk and Volmer, 2013). Participants were asked about their current gross income per month. There were 22 salary classes, starting at “no salary” and increasing in 500-euro steps up to “more than 10,000 euros” (Abele and Spurk, 2009). Promotions were assessed with an open question: “How many times have you been promoted so far in your career?” Number of promotions was thereby defined as any increase in hierarchical level and/or any considerable increase in job responsibilities or field of competence participants had experienced in their career (Van der Heijden et al., 2009).

Organizationally relevant outcomes were assessed using OCB and commitment. OCB was assessed with the German scale Fragebogen zur Erfassung des leistungsbezogenen Arbeitsverhalten FELA-S (Staufenbiel and Hartz, 2000). The scale comprises 25 items (e.g., “I take voluntary initiative in briefing new colleagues”). We used the subscale sportsmanship in our analysis. Participants answered the items on a 7-point scale ranging from 1 (*not at all true*) to 7 (*very true*).

Continuance (4 items) and affective (5 items) commitment were assessed with the commitment scale COMMIT (Allen and Meyer, 1990; Felfe and Franke, 2012). A sample item for continuance commitment is “I feel that I have too few options to consider leaving this organization” and for affective commitment is “This organization has a great deal of personal meaning for me.”

#### Career self-management factors

The CSM factors were assessed using the German version of the Career Futures Inventory (CFI; Rottinghaus et al., 2005; Spurk and Volmer, 2013). The inventory consists of 25 items, of which 11 assess career optimism (e.g., “I am eager to pursue my career dreams”), 11 assess career adaptability (e.g., “I can adapt to change in the world of work”), and 3 assess knowledge of the job market (“I'm good at understanding job market trends”). Participants answered all items on a 6-point scale ranging from 1 (*not at all true*) to 6 (*very true*).

#### Demographics

Participants reported their sex, age, academic degree, years of work experience, working time, and the size of their employer.

### Preliminary analysis

We made a confirmatory factor analysis using AMOS 22. We calculated composite reliability (CR), average variance extracted (AVE) scores and the square root of AVE scores by using the Stats Tool Package (Gaskin, 2012). Based on the results we computed our scale scores including the appropriate items (see the CFA model in the Appendix). The resulting model indices are a $\chi_{\left(941\right)}^{2}$ = 1390.81, *p* < 0.01, a CFI = 0.92, a RMSEA = 0.045 [0.04;0.05], and a SRMR = 0.06. As shown in the Tables A1, A2 in Supplementary Material we have convergent validity as shown by the AVE scores all above 0.50, we have reliability as shown by the CR scores all above 0.70, and we have discriminant validity based on the square root of the AVE scores being greater than any factor inter-construct correlations (Hair et al., 2010). The standardized regression weights of the CFA can be found in the Appendix (Tables A3, A4 in Supplementary Material).

To address common method (CM) bias we applied the marker-variable technique as described by Malhotra et al. (2006). As marker variable we used the smallest correlation among the variables as proposed by Lindell and Whitney (2001). We calculated the CM variance-adjusted correlations using Equation (1) (Malhotra et al., 2006, p. 1868). The results can be found in Table A5 in Supplementary Material (Appendix). We examined the significance of the CM variance-adjusted correlations using Equation (2) (Malhotra et al., 2006, p. 1868). The differences between the original and the CM variance-adjusted correlations were insignificantly small, indicating that the bias was not substantial.

### Analytic strategy

We used the methodology proposed by Preacher and Hayes (Preacher and Hayes, 2004, 2008) for mediation modeling to investigate the proposed hypotheses. This approach involves bootstrapping with 10,000 samples to obtain a point estimate of the indirect effect of the independent variable IP (*X*) on the dependent variables focusing on work-relevant outcomes (*Y*) through the mediating variables CSM factors (*MV*s) as well as bias-corrected 95% confidence intervals (CIs) for this estimate. In this analysis, several pathways are considered: pathway *c* (the total effect of *X* on *Y*), pathway *a* (*X* predicts *MV*), pathway *b* (*MV* predicts *Y*), and pathway *c*′ (the direct effect of *X* on *Y* when *MV* is controlled). The indirect effect of *X* on *Y* through *MV* is represented by the product of pathways *a* and *b* (*ab*). The PROCESS macro produced and offered by Hayes (2013) enables the calculation of all the pathways described above. Moreover, it enables the identification of an indirect effect even if *X* does not predict *Y* (Preacher and Hayes, 2004; Hayes, 2009).

## Results

### Bivariate correlations among the assessed constructs

The IP was negatively related to all indicators of employee-relevant subjective outcomes, namely, career satisfaction, job satisfaction, other-referent subjective career success, and internal and external marketability (*p*s < 0.05), thereby supporting Hypothesis 1. Regarding employee-relevant economic outcomes, the IP was significantly negatively related to salary and promotions (Hypothesis 2). Furthermore, we found the IP to be negatively related to OCB. Interestingly, we found no significant correlation of the IP to continuance (*r* = 0.05) or affective (*r* = −0.07) commitment (*p*s > 0.05). Hence, we found only partial support for our Hypothesis 3. Regarding the CSM factors, the IP was negatively related to career optimism, career adaptability, and knowledge of the job market. Moreover, the CSM factors were positively related to all indicators of employee-relevant outcomes, except for the relations between job satisfaction and internal marketability and knowledge of the job market, and between salary and optimism, which failed to reach significance. Regarding organizationally relevant outcomes, optimism and adaptability were positively related to OCB. Furthermore, adaptability was negatively related to continuance commitment and optimism correlated significantly with affective commitment in a positive direction.

These results indicated that it would be useful to investigate mediation analyses. Hence, we investigated the mediating role of the CSM factors in the negative relationship between the IP and the work-relevant outcomes.

### Mediating effects

To examine which CSM factors mediated the effects of IP on the work-relevant outcomes, we conducted mediation analyses using the PROCESS macro offered by Hayes (2013, Model 4). We calculated specific indirect effects using 10,000 bootstrap iterations. If the bias-corrected 95% CI does not include zero, the indirect effect is considered to be significant. We conducted one parallel mediation analysis for each indicator of employee-relevant (subjective and economic) and organizationally relevant outcomes. We used parallel multiple mediation modeling as it gave us the ability to compare the sizes of the indirect effects through the different CSM factors. Using this analysis strategy provided further insights concerning relevant mediating processes and enabled us to draw assumptions about relevant mediating CSM factors by looking at the specific indirect effects. Hence, we included all three CSM factors, namely, adaptability, optimism, and knowledge of the job market, in parallel. We report the effects in Table 3.

**Table 3:** **Mediating effects of CSM factors (MVs) in the relationship between the IP (X) and work-relevant outcomes (Y)**.

**Variable**			**Effects of the IP on *MV* (*a*)**	**Effects of *MV* on *Y* (*b*)**	**Total effect (*c*)**	**Direct effect (*c′*)**	**Indirect effect (*ab* paths)**	**95% CI**
***X***	***MV***	***Y***						
IP	Optimism	Career satisfaction	−0.43^***^	0.38^***^	−0.25^***^	−0.07	−**0.17**	**[−0.26, −0.09]**
IP	Adaptability		−0.34^***^	0.10			−0.03	[−0.09, 0.01]
IP	Knowledge		−0.39^***^	−0.03			0.01	[−0.02, 0.05]
IP	Optimism	Job satisfaction	−0.43^***^	0.31^***^	−0.34^***^	−0.22^**^	−**0.13**	**[−0.25, −0.05]**
IP	Adaptability		−0.34^***^	0.10			−0.03	[−0.10, 0.02]
IP	Knowledge		−0.39^***^	−0.11			0.04	[−0.00, 0.11]
IP	Optimism	Other-referent subjective career success	−0.43^***^	0.36^***^	−0.31^***^	−0.11^*^	−**0.20**	**[−0.25, −0.08]**
IP	Adaptability		−0.34^***^	0.06			−0.02	[−0.07, 0.02]
IP	Knowledge		−0.39^***^	0.07			−0.04	[−0.08, 0.00]
IP	Optimism	Internal marketability	−0.43^***^	0.20^**^	−0.26^***^	−0.15^*^	−**0.09**	**[−0.16, −0.03]**
IP	Adaptability		−0.34^***^	0.13^*^			−**0.05**	**[−0.11, −0.01]**
IP	Knowledge		−0.39^***^	−0.03			−0.01	[−0.03, 0.05]
IP	Optimism	External marketability	−0.43^***^	0.20^**^	−0.15^*^	0.09	−**0.09**	**[−0.16, −0.04]**
IP	Adaptability		−0.34^***^	0.30^***^			−**0.10**	**[−0.18, −0.04]**
IP	Knowledge		−0.39^***^	0.14^**^			−**0.06**	**[−0.12, −0.01]**
IP	Optimism	Salary	−0.43^***^	−0.23	−1.22^***^	−0.75^*^	0.10	[−0.14, 0.37]
IP	Adaptability		−0.34^***^	0.79^*^			−**0.27**	**[−0.55, −0.09]**
IP	Knowledge		−0.39^***^	0.79^**^			−**0.31**	**[−0.64, −0.10]**
IP	Optimism	Promotions	−0.43^***^	−0.03	−0.28^*^	−0.07	0.01	[−0.14, 0.10]
IP	Adaptability		−0.34^***^	0.30			−**0.10**	**[−0.26, −0.00]**
IP	Knowledge		−0.39^***^	0.25^*^			−**0.10**	**[−0.26, −0.00]**
IP	Optimism	Organizational citizenship behavior	−0.43^***^	−0.01	−0.50^***^	−0.44^***^	0.00	[−0.07, 0.07]
IP	Adaptability		−0.34^***^	0.31^**^			−**0.10**	**[−0.22, −0.04]**
IP	Knowledge		−0.39^***^	−0.09			−0.03	[−0.01, 0.11]
IP	Optimism	Continuance commitment	−0.43^***^	0.04	0.07	−0.04	−0.02	[−0.09, 0.05]
IP	Adaptability		−0.34^***^	−0.33^***^			**0.11**	**[0.05, 0.22]**
IP	Knowledge		−0.39^***^	−0.02			0.01	[−0.05, 0.06]
IP	Optimism	Affective commitment	−0.43^***^	0.20^**^	−0.09	−0.05	−**0.09**	**[−0.18, −0.02]**
IP	Adaptability		−0.34^***^	−0.08			0.03	[−0.03, 0.09]
IP	Knowledge		−0.39^***^	−0.05			0.02	[−0.03, 0.07]

N = 238. X, Independent variable; MV, mediating variable; Y, dependent variable; IP, impostor phenomenon; CI, confidence interval; 10,000 bootstrap samples were used. Knowledge = Knowledge of the job market.

* *p < 0.05*.

** *p < 0.01*.

*** *p < 0.001*.

Significant specific indirect effects are in boldface.

We found some support for Hypothesis 4 in our data, as the relation between the IP and all indicators of employee-relevant subjective outcomes was mediated by optimism, as indicated by the CIs that do not contain zero. Regarding career and job satisfaction as well as other-referent subjective career success, the results show that optimism was the only significant mediator. Finally, when examining the influence of the IP and optimism on career satisfaction concurrently, the effect of the IP was no longer significant (direct effect *c*), thereby indicating a complete mediation (Preacher and Hayes, 2004). In the relation of the IP and external marketability, knowledge of the job market emerged as a significant mediator in addition to optimism. The relation to external as well as internal marketability was additionally mediated by adaptability.

Regarding employee-relevant economic outcomes, results show that adaptability and knowledge of the job market mediated the effect of the IP on salary and promotions. As the bias-corrected 95% CIs for the indirect effects (*ab*) did not include zero, these mediating effects can be considered significant. The results further indicate that a partial mediation occurred, as the direct effect of the IP on salary (*c*′) diminished considerably but remained significant. On the subject of promotions, we found a complete mediation of adaptability and knowledge of the job market as the direct effect of the IP on promotions wasn't significant anymore. Hence, as postulated, an indirect effect of the IP on economic outcomes through adaptability and knowledge of the job market was found (Hypothesis 5).

As hypothesized (Hypothesis 6), adaptability was found to be a significant mediator, thereby indicating that the negative relation between the IP and OCB can be explained through reduced adaptability.

In considering the non-significant total effect of the IP on commitment, the IP was associated with continuance commitment only through adaptability and with affective commitment through optimism. However, mediation modeling clarified the relationship between the IP and commitment, as it was still possible to test for indirect effects in the absence of a significant *X*–*Y* relationship (Preacher and Hayes, 2004; Hayes, 2009). Hence, these results are in line with Hypotheses 7 and 8, even if the initial assumed total effect failed to reach significance. Interestingly, even if the total effect of continuance commitment was positive, the direct effect emerged as negative. All significant mediating effects are displayed in the Appendix (see Figures A1, A2 in Supplementary Material).

## Discussion

As predicted, we found the IP to be negatively related to employee-relevant subjective (Hypothesis 1) and economic outcomes (Hypothesis 2), as well as to one of the organizationally relevant outcomes (Hypothesis 3). Specifically, we replicated the negative relation between the IP and job satisfaction as well as on OCB that was reported in previous research as well (Grubb and McDowell, 2012; Vergauwe et al., 2015). Regarding commitment, we did not find a direct significant relation of the IP with affective commitment thereby supporting the findings of Vergauwe et al. (2015). However, we didn't either find one with continuance commitment as it was the case in previous studies (Grubb and McDowell, 2012; Vergauwe et al., 2015). Furthermore, we investigated if beside the direct effects, some indirect effects exist and inspected them using mediation analyses.

Regarding this mediating effects, career optimism emerged as a significant mediator in the negative relation of the IP and employee-relevant subjective outcomes (Hypothesis 4). Our results show that the IP's reduction of career optimism explains impostors' lowered career and job satisfaction. Regarding internal marketability, our findings support our theoretical assumption, as the negative relation was explained through less career optimism. However, adaptability also emerged as a significant mediator in this relationship. As impostors feel they have fewer adaptability resources and are less optimistic regarding their career, their perceived internal marketability diminishes. This could be another reason why impostors do not strive for a higher position within their company (Neureiter and Traut-Mattausch, 2016). In addition, less optimism was the most prominent mediator in the negative relation between the IP and other-referent subjective career success. Further to career optimism, the relation of the IP and perceived external marketability was additionally mediated by knowledge of the job market, suggesting that if external information is needed, job market knowledge functions as a pertinent factor. The perception of external marketability was further mediated by less adaptability. Previous research also found an effect of adaptability on perceived external marketability (Spurk et al., 2016), where more career adaptability led to a higher perceived external marketability and in turn to less career insecurity. Transferring these results to the IP and our findings, employees with more impostor feelings have fewer adaptability resources, which are needed to gain high perceived external marketability. The loss spiral will continue, as less perceived external marketability fosters career insecurity.

As a consequence, impostors experiencing high career insecurity try to stay at their current employment by showing higher continuance commitment. Even if we did not replicate the previously found direct relation between the IP and continuance commitment (Grubb and McDowell, 2012; Vergauwe et al., 2015), we found an indirect one through career adaptability (Hypothesis 7). This can be connected to the findings regarding career insecurity (Spurk et al., 2016). A path from the IP to career adaptability, from adaptability to perceived external marketability and from marketability to continuance commitment is conceivable. Therefore, in addition to the parallel multiple mediation analyses conducted herein, a serial mediation analysis could be interesting. An exploratory investigation of this path showed a significant indirect effect in the expected way (results are displayed in the Appendix, Figure A3 in Supplementary Material). The IP reduced career adaptability, which is positively related to perceived external marketability, which in turn reduces continuance commitment. This significant indirect effect is the first evidence supporting the theoretical consideration and should be researched in detail.

Reduced career adaptability as well as knowledge of the job market also mediated the relation between the IP and employee-relevant economic outcomes. The IP reduced career adaptability and knowledge of the job market (Hypothesis 5), which are needed to achieve a higher salary as well as more promotions. It is possible that impostors do not update their job market knowledge and do not display adaptability behavior because they do not plan to leave their current employer or position. This is in line with previous research showing that impostors have less clear career plans (Neureiter and Traut-Mattausch, 2016). As a consequence they do not show any intention to leave, which might even weaken their position in salary negotiations beyond the damage caused by the IP itself. These considerations could be investigated exploratively, too. Serial mediation analyses revealed that the IP reduced adaptability, which is needed for high internal marketability and in turn fosters higher salaries. Additionally, the IP reduced job market knowledge, which is needed for high external marketability and again fosters high salary. Results are displayed in the Appendix (see Figures A4, A5 in Supplementary Material). We also found a negative influence of the IP on promotions through reduced adaptability. Regarding this finding we would consider that impostors' supervisors who recognize their abilities—rather than they by themselves—would offer them promotions. However, the lowered adaptability resources will foster them to handicap themselves and do not make use of the job offers out of the fear of being exposed as impostors, as indicated by the indirect effect. This is in line with the findings that impostor feelings go along with self-handicapping (Ross et al., 2001; Cowman and Ferrari, 2002), as well as less career striving or motivation to take over a leading position within the company (Neureiter and Traut-Mattausch, 2016).

Regarding organizationally relevant outcomes, we found some support for our hypotheses. The negative relation between the IP and OCB was mediated by less adaptability, as predicted (Hypothesis 6). This is in line with our prior considerations that the impostors' reduced adaptability might prevent them from engaging in extra-role behaviors.

Regarding commitment, less career optimism emerged as a significant mediator in the relation of the IP and affective commitment. Hence, a pessimistic career prospect leads employees to be less affectively committed to their organization as predicted (Hypothesis 8). Even if the total effect did not reach significance, its direction was consistent with previous research, where a negative relation has been found (Grubb and McDowell, 2012).

### Limitations and future research

As our data are based on self-reports, we cannot rule out common-method bias (e.g., Podsakoff et al., 2003). This is especially important to consider given that impostors tend to downgrade themselves, which can lead to *underreporting effects* (Vergauwe et al., 2015). Hence, the negative association with other-referent subjective career success could also be explained by impostors underestimating their own career success and overestimating that of former or current colleagues. The same pattern could occur for internal and external marketability. Therefore, instead of relying solely on employee self-reports, future research could also use other sources for measuring internal and external marketability, such as human resources inventories (Eby et al., 2003). Also, the negative association with OCB requires attention regarding underreporting effects. As Vergauwe et al. (2015) suggested previously, this finding could be “partially the result of impostors discounting or minimizing any extra-role behaviors they engage” (p. 579). Hence, OCB could be evaluated through peer ratings or supervisor judgments of extra-role behavior to distinguish between true and underreporting effects.

We tried to address common method bias using the marker-variable technique (e.g., Malhotra et al., 2006). However, we did not identify a marker variable a priori. Instead we applied it *post hoc* using the smallest correlation among the variables (Lindell and Whitney, 2001). In future research a marker variable should be carefully identified before the start of data collection considering the selection criteria proposed by Williams et al. (2010) to address this issue.

Future research could also include additional employee-relevant outcomes such as work–life balance (Finegold and Mohrman, 2001). This could be especially interesting as recent research demonstrated an impact of the IP on work–family conflict (WFC) through emotional exhaustion (Crawford et al., 2016). The researchers based their investigation on the conservation of resources (COR) theory. They hypothesized “that individuals who experience the IP lack the initial resources needed to meet work demands and, thus, experience emotional exhaustion, which leads to WFC” (Crawford et al., 2016, p. 1). COR theory could also be used to look at our findings. It is conceivable that impostors need so many resources to maintain the status quo that they have no capacity left to invest in, for example, updating their job market knowledge, which would be needed to increase employee-relevant outcomes such as external marketability. Regarding COR theory, our parallel multiple mediation modeling could be useful for answering the call to identify those resources, such as career adaptability resources, that are used to attain certain goals, such as high income (Halbesleben et al., 2014). Future studies should extend this line of research by incorporating other important resources, such as perceived social and organizational support. In addition to the herein displayed mediating effects, such resources could play a moderating role. For example, Vergauwe et al. (2015) found that when social support was high, the negative relation between the IP and job satisfaction as well as OCB disappeared. Crawford et al. (2016) also found that the indirect effect of the IP on work–family conflict through emotional exhaustion was weaker when employees reported high perceived organizational support.

## Conclusions

In sum, our data suggest that the negative effects of the IP can be explained through reduced CSM factors: Reduced career optimism affects employee-relevant subjective outcomes such as job and career satisfaction or perceived internal marketability as well as organizationally relevant outcomes such as affective commitment. Consequently, our results suggest supporting emotion-based factors such as career optimism to prevent impostors from having lowered employee-relevant and organizationally outcomes. Reduced knowledge of the job market mediates the relation of the IP and employee-relevant economic outcomes such as salary and promotions, but also employee-relevant subjective outcomes if they are complemented by comparison to others. Thus, our results suggest fostering knowledge-based factors such as knowledge of the job market will improve impostors' perceptions of their external marketability. Reduced career adaptability affects several ability-based employee-relevant outcomes, such as internal and external marketability and salary and promotion, as well as organizationally relevant outcomes, such as continuance commitment and OCB. Our results suggest supporting adaptability-based factors, such as career adaptability, as these are needed for many outcomes that are pertinent for employees and organizations alike.

In conclusion, all CSM factors seem worthy of support as they help employees achieve fair pay and promotions, develop commitment, increase extra-role behavior, and grow into being internally as well as externally valued employees. Our findings offer new insights regarding how the IP functions in the world of work, identifying a valuable starting point for interventions. Although we distinguished between employee- and organizationally-relevant outcomes, fostering CSM factors does not only support the employees. One might be left with the impression that if impostors' CSM factors are not supported, organizations could have high-achieving employees who do not have to be paid very much. However, only satisfied and committed employees will perform best in the long term. Hence, human resources managers, career coaches, and counselors should use our findings to develop helpful interventions. They should identify effective ways to promote employee's CSM factors, as Koen et al. (2012) did regarding career adaptability and Spurk et al. (2015) did regarding career optimism, to profit from high-achieving employees for a long time.

## Author contributions

Both authors (MN, ET) substantially contributed to the conception and the design of the work as well as in the analyses and interpretation of the data. The first author MN prepared the draft, the second author ET reviewed it critically and gave important intellectual input. Both authors (MN, ET) worked for the final approval of the version that should be published. Both authors (MN, ET) are accountable for all aspects of the work in ensuring that questions related to the accuracy or integrity of any part of the work are appropriately investigated and resolved.

### Conflict of interest statement

The authors declare that the research was conducted in the absence of any commercial or financial relationships that could be construed as a potential conflict of interest.

## Footnotes

^1^The scale was used by permission from Dr. Pauline Rose Clance.
