# Supplementary material for: Inspecting the Dangers of Feeling like a Fake: An Empirical Investigation of the Impostor Phenomenon in the World of Work
Source: Front Psychol. 2016 Sep 27;7:1445. doi: 10.3389/fpsyg.2016.01445 (PMC5037221; doi:10.3389/fpsyg.2016.01445)
Supplement: Supplementary file 1 [file Appendix.pdf]

Appendix

Table A1.

Validity and reliability table

Factor	CR	AVE	MSV	MaxR(H)
External marketability	.79	.55	.26	.80
Impostor phenomenon	.90	.50	.14	.93
Optimism	.87	.50	.38	.96
Adaptability	.90	.57	.29	.97
Affective commitment	.90	.64	.16	.98
Career satisfaction	.89	.68	.38	.98
Continuance commitment	.74	.50	.11	.98
Job market knowledge	.77	.53	.18	.98
OCB	.99	.98	.12	.98
Internal marketability	.86	.76	.26	.98

Table A2.

Factor correlation matrix with square root of the AVE scores on the diagonal

**Factor**	External marketability	Impostor phenomenon	Optimism	Adapt-ability	Affective commitment	Career satisfaction	Continuance commitment	Job market knowledge	OCB	Internal marketability
External marketability	**.75**									
Impostor phenomenon	-.17	**.71**								
Optimism	.47	-.37	**.71**							
Adaptability	.51	-.37	.54	**.75**						
Affective commitment	.06	-.09	.20	.04	**.80**					
Career satisfaction	.47	-.31	.62	.36	.31	**.82**				
Continuance commitment	-.28	.08	-.12	-.30	.34	.01	**.71**			
Job market knowledge	.38	-.34	.31	.43	-.01	.15	-.16	**.73**		
OCB	.19	-.34	.20	.30	.09	.21	-.10	.11	**.99**	
Internal marketability	.49	-.29	.40	.33	.40	.51	-.07	.14	.18	**.87**

Table A3.

Standardized regression weights of the CFA

Item	Factor						
	Impostor feelings CIPS (Clance, 1985)	Career satisfaction CS (Greenhaus et al.,1990)	Internal marketability IM (Eby et al., 2003)	External marketability EM (Eby et al., 2003)	OCB FELA-S (Staufenbiel and Hartz, 2000)	Continuance commitment OCC (Felfe and Franke, 2012)	Affective commitment OCA (Felfe and Franke, 2012)
1 CIPS 4	.63						
2 CIPS 6	.81						
3 CIPS 7	.57						
4 CIPS 9	.56						
5 CIPS 13	.84						
6 CIPS 14	.72						
7 CIPS 15	.77						
8 CIPS 17	.76						
9 CIPS 18	.68						
10 CS 1		.90					
11 CS 2		.92					
12 CS 4		.86					
13 CS 5		.58					
14 IM 1			.84				
15 IM 2			.90				
16 EM 1				.74			
17 EM 2				.69			
18 EM 3				.80			
19 FELA-S 3					.77		
20 FELA-S 8					.54		
21 FELA-S 18					.77		
23 OCC 1						.72	
24 OCC 2						.82	
25 OCC 4						.55	
26 OCA 1							.78
27 OCA 2							.86
28 OCA 3							.86
29 OCA 4							.79
30 OCA 5							.71

Table A4.

Standardized regression weights of the CFA

Item	Factor		
	Adapability CFI (Spurk and Volmer, 2013)	Optimism CFI (Spurk and Volmer, 2013)	Knowledge CFI (Spurk and Volmer, 2013)
31 CFI 1	.68		
32 CFI 2	.74		
33 CFI 3	.73		
34 CFI 4	.80		
35 CFI 5	.83		
36 CFI 6	.79		
37 CFI 7	.68		
38 CFI 12		.66	
39 CFI 15		.70	
40 CFI 16		.66	
41 CFI 17		.70	
42 CFI 21		.76	
43 CFI 22		.63	
44 CFI 23			.85
45 CFI 24			.77
46 CFI 25			.54

Table A5.

Adjusted correlations for the main variables using marker-variable corrections

Variable	1	2	3	4	5	6	7	8	9	10	11	12	13	14
1 Impostor feelings	(.90)													
2 Career satisfaction	-.29***	(.88)												
	4.62[a]													
3 Job satisfaction	-.27***	.52***	–											
	4.27[a]	9.31[a]												
4 Other-referent subjective career success	-.34***	.61***	.34***	(.67***)										
	5.52[a]	11.78[a]	5.52[a]											
5 Internal marketability	-.28***	.46***	.41***	.40***	(.76***)									
	4.45[a]	7.92[a]	6.87[a]	6.67[a]										
6 External marketability	-.15*	.41***	.23***	.41***	.38***	(.79)								
	2.30[a]	6.87[a]	3.60[a]	6.87[a]	6.27[a]									
7 Salary	-.25***	.21***	.23***	.35***	.21**	.27***	–							
	3.93[a]	3.27[a]	3.60[a]	5.70[a]	3.27[a]	4.27[a]								
8 Promotions	-.13*	.25***	.16*	.24***	.19**	.23***	.53***	–						
	1.98[a]	3.93[a]	2.46[a]	3.76[a]	2.94[a]	3.60[a]	9.56[a]							
9 Organizational citizenship behavior	-.38***	.21**	.19**	.14*	.17**	.18**	.16*	.19**	(.74)					
	6.27[a]	3.27[a]	2.94[a]	2.14[a]	2.62[a]	2.78[a]	2.46[a]	2.94[a]						
10 Continuance commitment	.06	-.001	.11	-.002	-.05	-.23***	-.02	-.06	-.15*	(.73)				
	0.89[a]	0.02[a]	1.67[a]	0.00[a]	0.74[a]	3.60[a]	0.28[a]	0.89[a]	2.30[a]					
11 Affective commitment	-.08	.28***	.56***	.11	.35***	.02	.11	.07	.09	.33***	(.90)			
	1.20[a]	4.45[a]	10.34[a]	1.67[a]	5.70[a]	0.28[a]	1.67[a]	1.05[a]	1.36[a]	5.33[a]				
12 Optimism	-.36***	.60***	.37***	.57***	.37***	.39***	.13*	.13*	.20**	-.09	.19**	(.87)		
	5.89[a]	11.47[a]	6.08[a]	10.61[a]	6.08[a]	6.47[a]	1.98[a]	1.98[a]	3.10[a]	1.36[a]	2.94[a]			
13 Adaptability	-.34***	.37***	.23***	.36***	.29***	.44***	.27***	.27***	.31***	-.26**	.02	.46***	(.90)	
	5.52[a]	6.08[a]	3.60[a]	5.89[a]	4.62[a]	7.49[a]	4.27[a]	4.27[a]	4.97[a]	4.10[a]	0.28[a]	7.92[a]		
14 Knowledge of the job market	-.30***	.15*	.04	.28***	.12	.33***	.30***	.22**	.10	-.11	-.02	.25***	.38***	(.75)
	4.80[a]	2.30[a]	0.58[a]	4.45[a]	1.83[a]	5.33[a]	4.80[a]	3.43[a]	1.51[a]	1.67[a]	0.28[a]	3.93[a]	6.27[a]	

Note. $N = 238$ (57% female, 43% male). Entries in parentheses on the diagonal are Cronbach's alpha reliability coefficients.
[a]*t*-statistic calculated using Equation (2) (Malhotra et al., 2006, p. 1868)
$^{*}p < .05$. $^{**}p < .01$. $^{***}p < .001$.

Figure legends

Figure A1. Significant specific indirect effects of the impostor phenomenon on employee-relevant subjective (A) and economic (B) outcomes through self-management factors.

Figure A2. Significant specific indirect effects of the impostor phenomenon on organizationally relevant outcomes through self-management factors.

Figure A3. Results of serial mediation analysis showing that the impostor phenomenon reduced career adaptability, which is positively related to perceived external marketability and in turn reduces continuance commitment.

Figure A4. Results of serial mediation analysis showing that the impostor phenomenon reduces career adaptability, which is needed for high internal marketability and in turn fosters high salary.

Figure A5. Results of serial mediation analysis showing that the impostor phenomenon reduced job market knowledge, which is needed for high external marketability and in turn fosters high salary.

Figure A1.

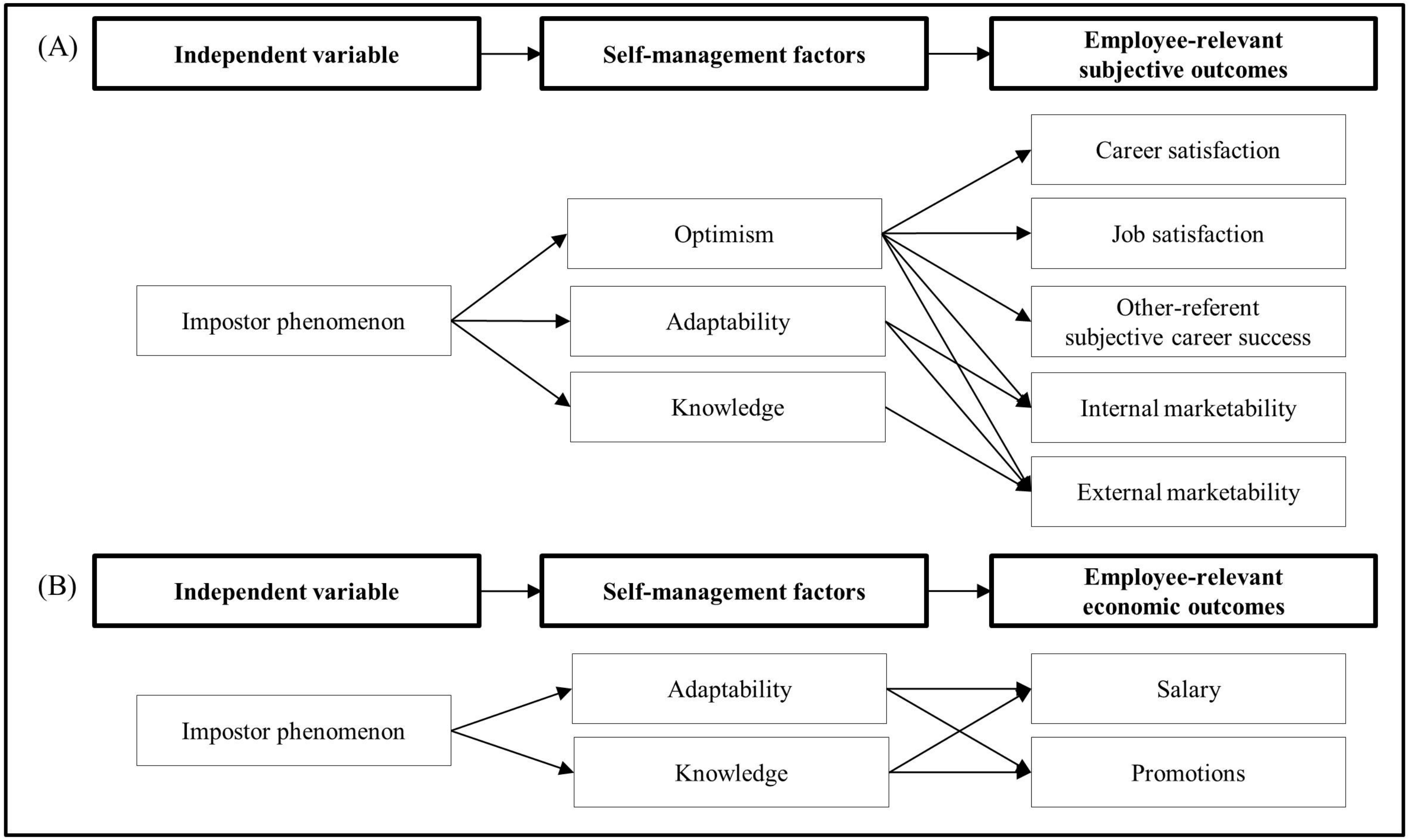
(A)
Independent variable
Self-management factors
Employee-relevant subjective outcomes
Career satisfaction
Optimism
Job satisfaction
Impostor phenomenon
Adaptability
Other-referent subjective career success
Knowledge
Internal marketability
External marketability
(B)
Independent variable
Self-management factors
Employee-relevant economic outcomes
Adaptability
Salary
Impostor phenomenon
Knowledge
Promotions

Figure A2.

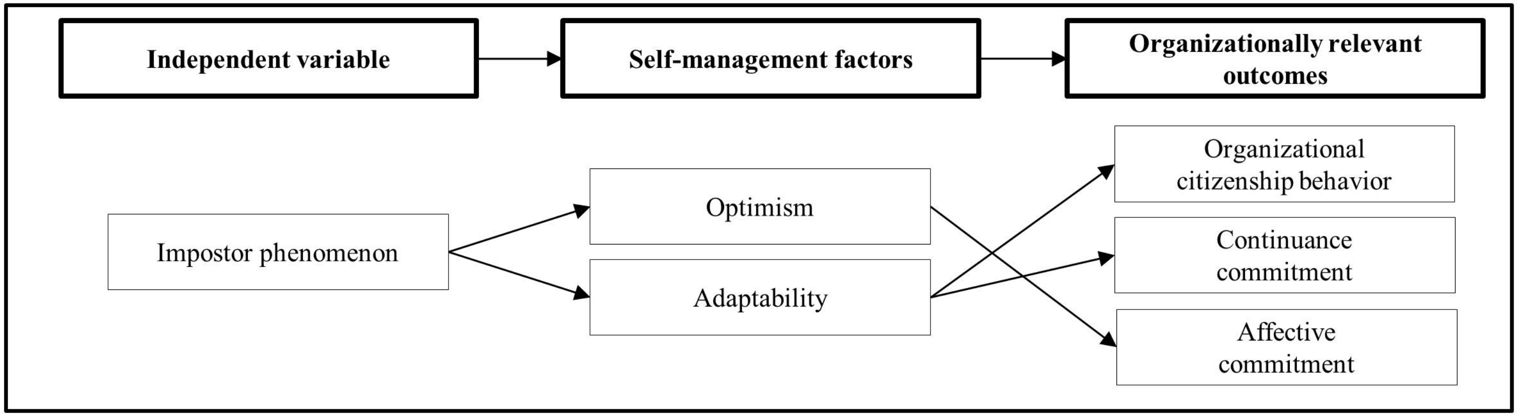

Independent variable
Self-management factors
Organizationally relevant outcomes
Impostor phenomenon
Optimism
Adaptability
Organizational citizenship behavior
Continuance commitment
Affective commitment

Figure A3.

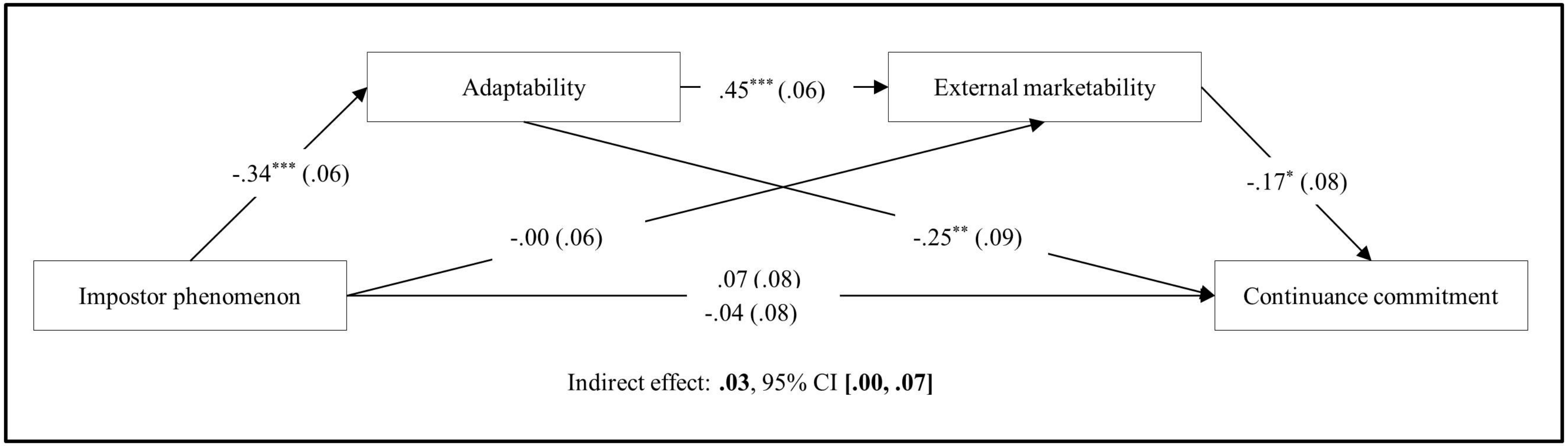
Adaptability
.45*** (.06)
External marketability
-.34*** (.06)
-.17* (.08)
-.00 (.06)
-.25** (.09)
Impostor phenomenon
.07 (.08)
-.04 (.08)
Continuance commitment
Indirect effect: .03, 95% CI [.00, .07]

Figure A4.

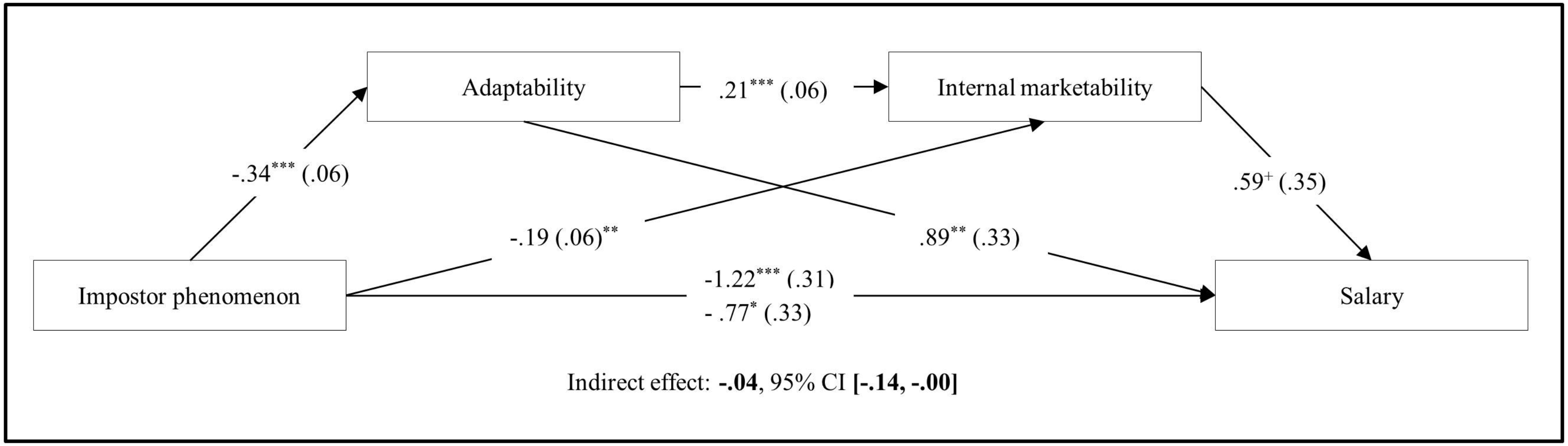
Adaptability
.21*** (.06)
Internal marketability
-.34*** (.06)
.59+ (.35)
-.19 (.06)**
.89** (.33)
Impostor phenomenon
-1.22*** (.31)
- .77* (.33)
Salary
Indirect effect: **-.04**, 95% CI **[-.14, -.00]**

Figure A5.

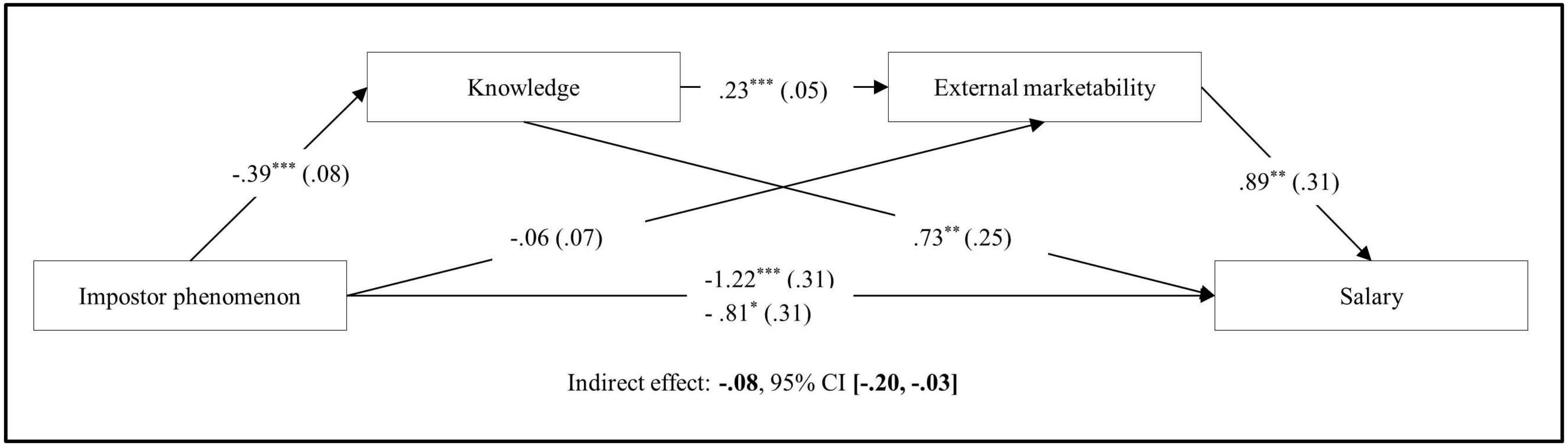
Knowledge
.23*** (.05)
External marketability
-.39*** (.08)
.89** (.31)
-.06 (.07)
.73** (.25)
Impostor phenomenon
-1.22*** (.31)
- .81* (.31)
Salary
Indirect effect: **-.08**, 95% CI **[-.20, -.03]**
